# Update on Physical, Psychological, and Quality of Life Management in Klinefelter Syndrome

**DOI:** 10.1210/clinem/dgaf261

**Published:** 2025-05-02

**Authors:** Brien Mehmet, Andrew A Dwyer, Channa N Jayasena, Steve Gillard, Sofia Llahana

**Affiliations:** School of Health & Medical Sciences, City St George's, University of London, Northampton Square, London EC1V 0HB, UK; Department of Endocrinology and Diabetes, Royal Free Hospital NHS Foundation Trust, London NW3 2QG, UK; Endocrinology, Diabetes, and Metabolism Service, Massachusetts General Hospital, Boston, MA 02114, USA; Harvard Reproductive Endocrine Sciences Center and Reproductive Endocrine Unit, Massachusetts General Hospital, Boston, MA 02114, USA; Department of Investigative Medicine, Imperial College London, Hammersmith, London W12 0NN, UK; School of Health & Medical Sciences, City St George's, University of London, Northampton Square, London EC1V 0HB, UK; School of Health & Medical Sciences, City St George's, University of London, Northampton Square, London EC1V 0HB, UK

**Keywords:** quality of life, Klinefelter syndrome, clinical guidelines

## Abstract

**Context:**

Klinefelter syndrome (KS), most commonly arises from a 47,XXY karyotype. While KS affects around 1 in 450 to 600 male births, an estimated 50% to 75% of cases go undiagnosed. Individuals with KS are at increased risk of metabolic, cardiovascular, and reproductive comorbidities, increasing mortality risk, psychological burden, and significantly diminished health-related quality of life (HR-QoL) compared to healthy controls. We provide an updated review of the recent literature on the clinical management for people with KS, associated comorbidities, and the implications on HR-QoL.

**Evidence Acquisition:**

A comprehensive literature search was conducted of key databases MEDLINE, CINAHL, Cochrane, Psychinfo, and EMBASE, followed by a gray search of relevant key papers in KS with medical guideline organizations being searched online and, where available, their publications and proposed guidelines being assessed. All databases were searched from their inception until December 2024, and English-language restrictions applied.

**Evidence Synthesis:**

Current evidence highlights the need for early detection, the importance of appropriate medication management, and multidisciplinary care to address infertility, cancer risks, neurocognitive deficits, and adverse mental health. Lifespan-specific interventions remain underexplored, necessitating further research to optimize outcomes and refine clinical guidelines.

**Conclusion:**

KS management is guided by only one endorsed guideline. International and interprofessional collaboration is needed to develop consensus documents. Existing guidelines pay minimal attention to the numerous HR-QoL challenges, overlooking the effect and diagnosis of psychological comorbidities. Enhancing HR-QoL and optimizing physical and mental well-being should be prioritized to improve HR-QoL outcomes in people with KS.

Klinefelter syndrome (KS) is the most common male chromosome aneuploidy, affecting around 1 in 450 to 600 male births. KS is thought to be caused by nondisjunction wherein normal cell division is disrupted, resulting in aneuploidy (XXY) or mosaicism. The cause of nondisjunction is unknown and likely affects all population groups (1).

KS is characterized by the addition of one or more X chromosomes (XXY). The prevalence of KS is likely to be much greater than reported, as an estimated 50% to 75% of men with KS remain undiagnosed (2-4). Almost 90% of men with KS have an XXY karyotype, and the remaining 10% mosaic KS (46,XY/47,XXY), while higher-grade aneuploidies (48,XXXY; 49,XXXXY) are routinely not described as KS due to the often more adverse phenotype (4).

The additional X chromosome directly affects genetic and cellular processes, particularly those related to reproductive development. It is believed most men with KS will experience testicular dysgenesis wherein gonads undergo hyaline degeneration resulting in small, firm testes. Currently, it is unclear when degeneration becomes apparent. However, research has shown a rapid decline in testicular health and function following the initial stages of puberty, leading to hypogonadism and poor development of secondary sexual characteristics (5-7). Genomic and epigenetic causes of this are believed to result from inactivation of the additional X chromosome and androgen receptor polymorphisms as CAGn length is negatively associated with testicular volume and greater atrophy (8, 9).

While this molecular-level testicular dysgenesis may not be visible daily, it can manifest physiologically and psychologically causing an array of adverse health-related quality of life (HR-QoL) outcomes and comorbidities (10, 11).

Common comorbidities in KS include hypogonadism, infertility, metabolic syndrome, cardiovascular disease, diabetes, osteoporosis, and an increased risk of mortality (12). Mental health disorders, such as depression and anxiety, are also prevalent.

Our previous systematic review demonstrated significant negative associations between HR-QoL and individuals with KS, with deficits observed across all tested domains compared to healthy controls (HCs) and population averages. This included a vast array of adverse psychological, psychosocial, and physical outcomes. Meta-analysis demonstrated reduced IQ scores when tested against controls. Twelve of the 13 studies suggested negative associations on all domains of HR-QoL, emphasizing the importance of understanding HR-QoL in people with KS and the relation between managing their health and well-being needs (13).

There have been several comprehensive reviews on the management strategies and recommendations for individuals with KS, highlighting key areas such as genetic mechanisms, clinical phenotypes, hypogonadism, and neurocognitive features (4, 14-16). In recent years there have been substantial advancements in understanding the condition, leading to the first international consensus document for the management of KS (17), providing multidisciplinary recommendations on diagnosis, management, and long-term health monitoring within KS. Additionally, large cohort studies have expanded knowledge of the associated comorbidities, including increased risks of neoplasia, metabolic diseases, osteoporosis, and cardiovascular disease (18, 19), emphasizing the importance of timely diagnosis and comprehensive management. Emerging research is also beginning to investigate potential associations between people with KS and degenerative diseases, aging, and the effect of KS throughout the life course (20).

We conducted a scoping review to evaluate current management guidelines, recommendations, and research on the clinical management of people with KS, while synthesizing findings on HR-QoL outcomes from a developmental perspective, contextualizing the effect of KS across different life stages. This approach offers new insights into KS management and identifies opportunities to improve HR-QoL outcomes.

## Methods

### Research Question/Objectives

This scoping review aims to provide a concise, up-to-date synthesis of the approach to managing KS. The review was conducted through an extensive literature search followed by a thematic synthesis, combining management guidelines, recommendations, and recent research across the subdomains of physical health, mental well-being, and HR-QoL. The literature search was completed using an a priori thematic framework to ensure critical areas of health affected in KS were included. Health domains were chosen from key literature on KS.

The research objectives are the following:

1. Highlight the current management and treatment guidelines for individuals with KS in the key health domains described.
2. Discover how current management guidelines address HR-QoL deficits.
3. Identify future areas for investigation that have the potential to reduce health inequalities and improve HR-QoL outcomes in individuals with KS.

### Identifying Relevant Studies

The databases MEDLINE, CINAHL, Cochrane, Psychinfo, and EMBASE were searched. A gray literature search followed, including key paper references and online searches of medical guideline organizations; where available, their publications and proposed guidelines were assessed, including the European Society of Endocrinology, the National Institutes of Health (USA), the National Institute for Health and Care Excellence (NICE, UK), the national health service (UK). All databases were searched from their inception until December 2024.

### Eligibility Criteria

All empirical studies, emerging research, and relevant literature involving clinical guidelines and the management of people with KS, or the chosen health domains were assessed for inclusion. No publication date restrictions were imposed. See supplementary material (21) for inclusion/exclusion criteria.

### Study Screening and Selection

**Figure 1.**
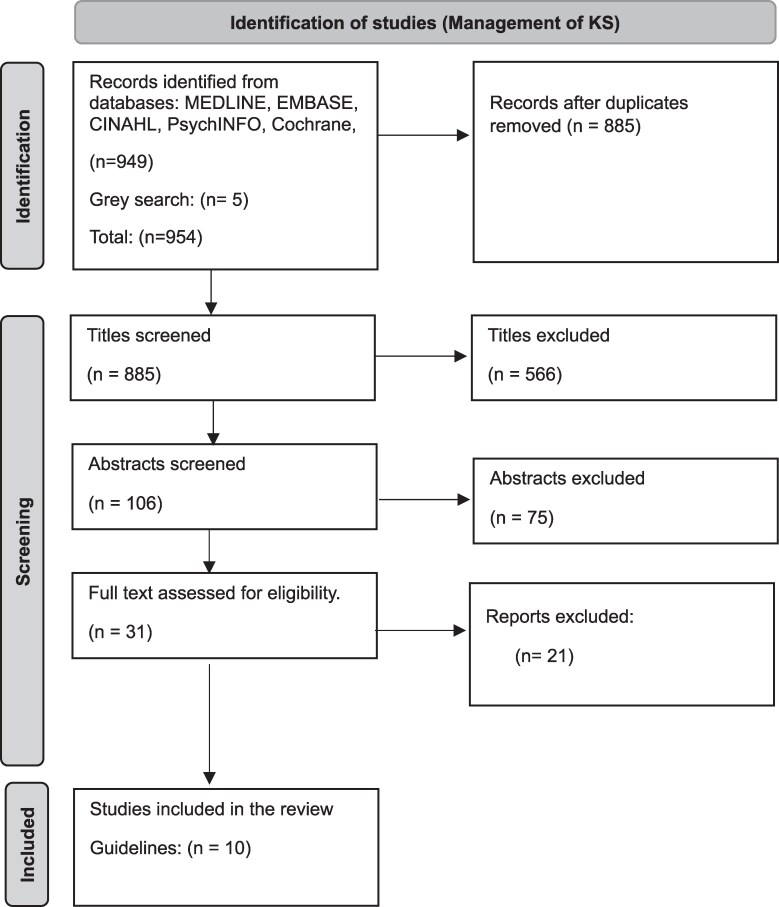
PRISMA flowchart of study search, screening, and selection (management of Klinefelter syndrome [KS]). Flowchart describing the selection of literature process within the scoping review for studies, guidelines, and literature on the clinical management of people with KS.

The eligibility assessment was performed as a blind independent review by 2 authors (B.M. and S.L.); all disagreements were resolved by consensus and did not require a third reviewer. The PRISMA flow diagram (22) (Fig. 1) shows the literature selection process for KS management guidelines.

## Results

### Data Synthesis

A thematic synthesis was completed by analyzing the key chosen themes, namely: “diagnosis, metabolic disorders, and body composition, cardiovascular and thrombosis risk, bone disorders, neoplasia, fertility management and treatment, testosterone (T) replacement therapy, psychological health and neurocognitive function, and KS through the lifespan.” The chosen themes were selected on an a priori basis, reflecting the most prominent health domains effected. These areas were also dominant in current key literature (4, 23).

### Characteristics of Included Studies

From the initial search, 949 records were identified, of which 10 met the inclusion criteria, providing guidelines on treating and caring for people with KS. These studies covered both general and domain-specific management areas. Table 1 presents a summary of the characteristics extracted from each study providing insight into management approaches. Further studies were included that provided insight into the key domains of health mentioned, but they were not management guidelines.

**Table 1.** Summary of included studies (management of Klinefelter syndrome). Identifying included subthemes

Reference	Diagnosis	Metabolic	Cardiovascular	Bone disorders	Neoplasia	Fertility	TRT	Psychological health	Transition
Aksglaede et al, 2013 (24)	Yes	Yes	Yes	Yes	Yes	Yes	Yes	Yes	—
Groth et al, 2013 (16)	—	Yes	—	Yes	Yes	Yes	Yes	Yes	—
Zitzmann et al, 2021 (17)	Yes	Yes	Yes	Yes	Yes	Yes	Yes	Yes	Yes
Nieschlag et al, 2016 (25)	Yes	—	—	—	—	Yes	Yes	Yes	—
Salzano et al, 2016 (38)	—	—	Yes	—	—	—	—	—	—
Kailash et al, 2021 (54)	—	—	—	—	—	Yes	—	—	—
Jayasena et al, 2022 (66)	—	—	—	—	—	—	Yes	—	—
Garolla et al, 2024 (27)	Yes	—	—	—	—	—	—	—	—
Martin et al, 2023 (96)	—	—	—	—	—	—	—	Yes	—
Grande et al, 2023 (42)	—	—	—	Yes	—	—	—	—	—

Abbreviation: TRT, testosterone replacement therapy.

## Thematic Synthesis

### Diagnosis

The current consensus for diagnosing KS in suspected patients is to use microarray or karyotyping analysis (17, 24, 25). These methods are the gold standard for confirming KS, as they directly detect chromosomal anomaly. More severe chromosomal aneuploidies (XXXY or greater) are typically linked to worse phenotypic outcomes. However, despite these diagnostic tools' availability, the average diagnosis age remains notably late, around 25 years (26, 27). This delay is partly due to the lack of widespread implementation of newborn screening or noninvasive prenatal screening, which could potentially improve early detection rates. The limited early screening methods likely contribute to the lack of timely diagnosis. It is plausible that only the most severely affected boys are diagnosed early, when symptoms related to speech, language, and the associated psychosocial challenges prompt further investigation. In approximately 75% of cases, diagnosis does not become evident until later in life, typically due to infertility. Empirical studies have shown adult patients with KS to exhibit significantly greater autism spectrum disease symptoms and various adverse behavioural traits (depression, anxiety, social exclusion, communication skills, attention-deficit hyperactivity disorder) when compared to HCs (28-31). Research completed on a US military veteran population found that clinical diagnosis of KS, even if delayed, was associated with reduced medical morbidity (19), speculated to be due to increased hospital appointments managing various comorbidities.

Further research by Garolla et al (27) underscores the importance of a multidisciplinary approach in diagnosing and managing KS, including collaboration among psychologists, geneticists, and endocrinologists and emphasizes the importance of nuanced communication both from endocrinological and psychological experts (27). However, evidence supporting this remains limited. The broader adoption and effect of such strategies require further validation.

### Metabolic Disorders and Body Composition

People with KS are at an increased risk of developing metabolic syndromes and insulin resistance due to the chromosomal structure and resulting hypogonadism (16, 17). Additionally, KS is associated with increased fat mass and reduced lean muscle mass (17, 24, 32). Boys and adolescents experience androgen deficiency leading to below-average-sized penis and testes, with additional phenotypes including decreased muscle tone (hypotonia) (33).

Zitzmann et al (17) recommend educating patients on lifestyle modifications to mitigate metabolic risk and rationale for assessing body composition, fasting glucose, glycated hemoglobin A_1c_, and lipids. These measures are crucial for the timely detection of metabolic disease and guiding interventions to prevent further metabolic disruption (ie, type 2 diabetes [T2DM]). Research has shown T2DM to be associated with poorer overall HR-QoL in a non-KS population (34, 35). Systematic review and meta-analysis identified factors that negatively affect HR-QoL in patients with T2DM, including “anxiety, depression, reduced physical activity, insulin use, diets high in red meat, hypertension, and duration of diabetes” (36).

### Cardiovascular and Thrombosis Risk

Three guidelines for managing and treating people with KS note the need to monitor and treat cardiovascular disease due to the increased risk associated with KS (17, 24, 25). A large British cohort study (n = 3518) from 1959 to 2003 (37) demonstrated increased overall mortality in people diagnosed with KS. The elevated mortality rate highlighted cardiovascular disease as a key factor (standardized mortality rate: 1.3; 95% CI, 1.1-1.5). Current management guidelines include at least one 12-lead electrocardiogram for all people with KS. Salzano et al (38) also recommends a “complete cardiovascular workup” in KS, including an algorithm for assessing preclinical and clinical risk.

There are 2 main hypotheses regarding the cause of cardiovascular abnormalities in people with KS. First, some posit that hypogonadism is the main driver of cardiovascular involvement in KS. Research identifies reduced fibrin clot lysis, increased fibrinogen levels, factor XIII, and plasminogen activator inhibitor type 1 in KS, contributing to an increased prothrombotic state. This association is hypothesized to be driven by the ongoing hypogonadism (39). Further research detected a plasma procoagulant imbalance in KS, highlighting increased thrombin generation in part driven by increased levels of factor VIII, presenting a significantly increased risk of clotting and thrombosis in KS (40). The current reason for increased factor VIII levels in people with KS is unclear.

While hypogonadism is seen to play a role in the cardiovascular risk associated with KS, others point to the underlying genetic architecture (ie, aneuploidy) as the cause of worse cardiovascular profiles. There are plausible arguments for both positions, with no consensus at present. Pasquali et al (41) compared people with KS to hypogonadal men with a normal chromosomal complement receiving T replacement therapy (TRT). Investigators observed that cardiovascular abnormalities resolved in HCs but not in people with KS. These data suggest that genetic architecture, as opposed to hypogonadism per se, underlies cardiovascular abnormalities in KS. If true, this implies cardiovascular involvement is a nonmodifiable aspect of KS.

There is a strong scientific justification for both hypotheses, suggesting that these mechanisms may be concurrently operative, each contributing to the increased cardiovascular risk observed in individuals with KS.

Disease-specific guidelines for identification, management, and treatment could improve patient outcomes by controlling cardiovascular comorbidities in people with KS.

### Bone Disorders

Impaired bone health, including low bone mineral density (BMD), manifests as osteopenia/osteoporosis, often leading to fractures in men with KS. Current KS guidelines recommend following European Academy of Andrology clinical guidelines on managing bone health in the androgenic outpatient clinic, including dual-energy x-ray absorptiometry (DXA) and monitoring serum vitamin D levels (17). Additional research conducted by Grande et al (42) offers several clinical suggestions for the diagnosis and treatment of bone health in KS. They propose conducting DXA scans for all hypogonadal men, including those with KS. Grande and colleagues emphasize that BMD alone accounts for only 60% to 70% of the assessments of bone health conditions. Hence, further evaluations of bone strength and fracture risk beyond BMD through DXA scans are recommended. Recommendations also include the use of trabecular bone score evaluation (43) and established methods of sarcopenia diagnosis (44, 45).

The recommended treatment options were vitamin D supplementation (calcifediol rather than cholecalciferol) with investigation of 25-hydroxyvitamin D levels, TRT in hypogonadism, and lifestyle changes (smoking cessation, reduced alcohol intake, and exercise) (42).

Research demonstrates that decreased BMD is not solely associated with low serum T levels but is associated with muscle strength, adequate TRT, and bone markers (46). Furthermore, it has been shown that young men with rare variants in receptor for Leydig insulin-like peptide (INSL3) (RXFP2) exhibit reduced bone mass. Ferlin et al (47) hypothesized that reduced bone mass in KS may be related to low circulating concentrations of INSL3, yet this postulation remains speculative without robust supporting empirical data. Further research illuminates the negative relationship between high follicle-stimulating hormone (FSH) levels and diminished BMD, particularly at the spine. The duration of elevated FSH levels is believed to contribute to the negative association.

Di Nisio et al (48) examined associations between sclerostin and INSL3 (the negative regulator of bone formation in osteocytes) in human osteocytes isolated from participants with KS. Investigators observed a negative association between INL3 and sclerostin. Notably, sclerostin was significantly increased in KS patients. However, INSL3 reduced sclerostin expression, further implicating INSL3 in KS-associated bone disorders. Several factors affect bone health in male patients with KS, displaying the need for further investigation to provide evidence-based guidelines.

### Neoplasia

The recommendations for management and identification of neoplasia are included within 2 studies/guidelines (17, 24) highlighting the need for breast examination due to the increased risk of breast cancer in men with KS. Although research is limited, there are case reports and a recent review indicating people with KS also have an increased risk of extragonadal germ cell tumors (49). Research emphasizes that male patients with KS are more likely to be diagnosed with a mediastinal extragonadal germ cell tumor (50), suggesting a low threshold for radiologic examinations to ensure timely diagnosis. Furthermore, Ji et al (51) conducted a national database study (Sweden) using standardized incidence ratios (SIRs), concluding that patients with KS had a greater risk of developing hematological malignancy (SIR = 2.72), non-Hodgkin lymphoma (SIR = 3.02), breast cancer (SIR = 4.37), and leukemia (SIR = 3.62). A study by di Fraia et al (52) found that men with KS had a significantly higher prevalence of nodular thyroid disease than HCs (*P* = <.001), hypothesized to be due to low levels of free thyroxine (FT4), inappropriate thyrotropin secretion, and/or genetic instability. Previous research found lower FT4 concentrations in adults with KS compared to HCs (*P* = .001), further illuminating the relationship between the gene dosage effect and the extent of aneuploidy on thyroid function by finding a progressive decrease in FT4 levels as the number of supernumerary Xs increased (53). Reassuringly, all identified nodules were cytologically benign. Research into the prevalence of thyroid nodules, thyroid cancers, and their management would be beneficial.

### Fertility Management and Treatment

KS is characterized by primary testicular failure resulting in hypergonadotropic hypogonadism with elevated luteinizing hormone and FSH levels, and low T. Due to the association between KS and hypogonadism, patients often present in infertility clinics due to unsuccessful attempts at conception and are subsequently diagnosed with KS. In doing so, a detailed semen analysis is collected to determine whether sperm collection may be feasible if healthy spermatozoa are present in the ejaculate. Azoospermia can be managed by using a preexisting protocol (54).

Advances in fertility treatment and procedures such as microsurgical testicular sperm extraction (micro-TESE) mean people with KS are no longer considered infertile; a meta-analysis of 37 trials (n = 1248; mean age 30.9 ± 5.6 years) showed that the surgical sperm retrieval rate per TESE cycle was achieved in almost half of the men with KS (44%; 95% CI, 39%-48%) (55). Although retrieval of spermatozoa is possible, evidence for a take-home baby rate following micro-TESE and female-assisted reproductive treatment is still limited. Surgical outcomes should be optimized by identifying appropriate surgical candidates and hormonal manipulation (56). Further predictors of surgical success would be testicular volume, cryptorchidism history, and inhibin B, a biomarker that correlates with testicular volume and sperm count (57).

Sperm cryopreservation is an option for patients, used for the storage of sperm when required for fertility treatments. It can also be used as a safety net for patients with testicular hypogonadism following treatment to induce spermatogenesis, or prior to certain surgical interventions/gonadotoxic or gonadal-suppressive treatments (57). Sperm samples can be retrieved during micro-TESE and stored for future use. It is important to note many strict regulations, policies, and testing procedures are required to store sperm and use them for fertility treatments successfully; patients should be consulted on these to ensure that they can give informed consent.

### Testosterone Replacement Therapy

KS is the most common congenital cause of primary male hypogonadism. Individuals with the classical phenotype (XXY) typically exhibit diminished testicular size, function, and impaired spermatogenesis, though this presentation varies, especially in cases of mosaicism (58). Testicular dysfunction results from progressive gonadal failure, characterized by tubular atrophy, sclerosis, and maturation arrest, leading to fibrosis and hyalinized tissue due to germ and Sertoli cell loss. Histological examination reveals apparent Leydig cell hyperplasia due to the loss of virtually all Sertoli cells (59, 60), as well as interstitial tissue proliferation (61). Testicular volume in KS patients rarely exceeds 5 to 6 mL (16), with degeneration becoming most apparent at the cusp of puberty, when testicular function and architecture begins to decline (62).

T levels vary considerably, particularly in younger individuals and those with mosaicism (46,XY/47,XXY). While most men with KS have subnormal total T levels, approximately 20% to 30% maintain total T within the low-normal range, particularly in milder cases or mosaic forms (63). Elevated luteinizing hormone is observed in more than 80% of adult KS patients, even in patients with normal or low total T levels, indicating compensated hypogonadism (62, 64). One study investigating different forms of hypogonadism in men with KS found 15.6% had normal total T values at presentation, 51.4% had compensated hypogonadism, and 33% had primary hypogonadism (65)

Following clinical suspicion, primary hypogonadism should be biochemically confirmed, with TRT being the primary treatment (66). Correct administration and adherence to TRT can improve muscle mass, strength, mood, libido, emotional well-being, and BMD (67, 68). Patient-clinician discussions are crucial for optimal adherence and treatment choice. Recent literature has identified that topical gels generally have poorer adherence than subcutaneous injections (69, 70). Current guidelines for TRT provide clear evidence for the indications, use, and monitoring of male hypogonadism and TRT (71).

Men with KS are associated with a proembolic phenotype due to comorbidities (diabetes, hypertension, and hypercholesterolemia). Current evidence on TRT and thrombotic risk is conflicting. Earlier studies suggested that initiating TRT is linked to an increased risk of thromboembolism, peaking within 6 months before subsiding (72). However, recent meta-analyses, which offer substantial evidence, indicate that TRT does not increase the risk of arterial thrombotic events and provide inconclusive data on deep vein thrombosis due to limited randomized controlled trial data (73). A recent cohort study found no increased thrombotic risk with TRT in patients with KS, showing lower rates of venous thromboembolism and thrombotic deaths than in untreated people with KS (74).

Research highlights 5 key outcomes important to men with hypogonadism: symptoms' effect on daily life, serum T levels, access to treatment information, perceived TRT effects, and treatment preferences (75). People with KS frequently experience a broad spectrum of hypogonadism-related symptoms. Despite this, they show considerable adaptability and resilience, often effectively using problem-focused coping strategies (76). This can make identifying and assessing symptoms challenging, as men with KS may develop sophisticated methods to handle their difficulties while masking the extent of their experiences.

Considering these factors can improve HR-QoL and patient outcomes during TRT. Hypogonadism often negatively affects daily functioning, energy, sexual ability, and cognitive functions, and affects self-perceived masculinity (77-79). Adult men with KS report reduced sexual life satisfaction, sexual desire, and negative scores on the International Index of Erectile Function scale (28, 30, 80-82). Sexual function, mood, and emotional well-being have all been shown to improve in men with KS when treated with TRT (74). However, the effect of TRT on overall HR-QoL in men with KS has yet to be investigated.

### Psychological Health and Neurocognitive Function

Psychological comorbidities associated with KS include anxiety, depression, autism spectrum disorder symptoms, psychosis, reduced intelligence, attention span, and neurocognitive disorders such as cognitive function (28, 80, 83-87). However, symptom variability is considerable, with no distinct psychological phenotype being identified in people with KS. Negative associations between psychological symptoms and HR-QoL in people with KS have been identified (13).

There are several hypotheses concerning the increased risk of cognitive and psychological disorders in people with KS. Studies have identified neuroanatomical changes, including significant differences in total brain, gray, and white matter volume compared to the general population (88-90). Gray and white matter volume were reduced in the insula, putamen, caudate nucleus, hippocampus, amygdala, right parahippocampal region, cerebellum, temporal lobe, and frontal inferior orbital (91, 92), with widespread alterations in global functional connectivity among the inferior frontal gyrus, temporal-parietal area, and hippocampus in boys aged 8 to 17 years with KS (93). Table 2 presents effect sizes, highlighting the distinct neuroanatomical differences between participants with KS and HCs.

**Table 2.** Calculated effect sizes on the neuroanatomical differences in people with Klinefelter syndrome

Reference	Measure	Participants (n)	*P*	*t*	Effect size, Cohen *d*
(88) Bryant et al, 2011	TGMV	KS (n = 31) HCs (n = 36)	.072	−1.827	.44
	TWMV		.059	−1.919	.47
	TGMV + TWMV		.061	−1.904	.46
(89) DeLisi et al, 2005	Intercranial contents (ANCOVA)	KS (n = 11) HCs (n = 11)	.05	2.0761	.89
(91) Skakkebæk et al, 2014	TGMV, mL	KS (n = 65) HCs (n = 65)	<.01	3.08	.54
	TWMV, mL		<.01	1.15	.51
(92) Warwick et al, 1999	Whole-brain volume, mL	KS (n = 10) HCs (n = 25)	< .01	4.4104	1.64
(93) Li et al, 2024	Regional brain activity	KS (n = 46) TDs (n = 51)			
	fALFF				
	L precentral gyrus		.002	3.641	.74
	L postcentral gyrus		.001	3.696	.75
	R precentral gyrus		.001	3.962	.80
	R postcentral gyrus		<.001	4.226	.86
	R supplementary		<.001	5.053	1.02
	ReHo				
	L caudate		<.001	−5.110	−1.04
	R caudate		<.001	−4.899	−.99

Abbreviations: ANCOVA, analysis of covariance; fALFF, fractional amplitude of low-frequency fluctuations; HCs, healthy controls; KS, Klinefelter syndrome; L, left, R, right; ReHo, regional homogeneity; TDs, typically developing boys; TGMV, total gray matter volume; TWMV, total white matter volume.

It is understood that these changes are associated with functional impairment and language abnormalities as well as vulnerability to schizophrenia, psychosis, autism spectrum disease, mood, and anxiety disorders. Continued research is required to further understand the relationships between brain structure alterations and neurocognitive outcomes in people with KS.

A cohort study (n = 218) found that only 16% of participants with KS had received psychological support during childhood and 29% during adulthood, highlighting a substantial gap between the psychological support received and the subjective need for such support, and underscoring the necessity of offering psychological support (94).

Clinical guidelines for people with KS recommend monitoring psychological illness and using a multidisciplinary approach involving psychologists, genetic counseling, and speech and language therapists (16, 17). Adverse psychiatric health is often associated with reduced HR-QoL. Yet, the extent of this effect remains unclear. Existing HR-QoL measures may not ask the condition-specific questions unique to people with KS (95).

Identifying and effectively using care pathways for psychiatric comorbidities may improve patient outcomes. Martin et al (96) explored an innovative neurocognitive-behavioral treatment program designed to address specific vulnerabilities associated with KS. The new social management guideline focused on addressing executive dysfunction and tailoring interventions to behavioral and cognitive characteristics specific to KS. When used, this was shown to be a promising and effective approach for enhancing self-control and social adaptation.

The identified guidelines emphasize the importance of psychological intervention when there are clinical concerns, but there is limited guidance on assessing the psychiatric profile of patients with KS. As a result, psychiatric and HR-QoL issues for patients with KS may not be adequately addressed. This could be attributed to a multitude of factors, such as “limited clinical consultation time, lack of awareness about the condition, and a shortage of psychiatric guidelines for treating mental health in KS.”

### Klinefelter Syndrome Through the Lifespan

KS can negatively affect HR-QoL at all stages of life. Clinicians and guidelines should focus on interventions to minimize impairments. Although there is no apparent phenotype or distinct developmental profile for children with KS, research shows several areas that experience developmental delays, such as “speech, motor skills, language, cognitive function, behaviors (including social behaviors), testicular development/function, and growth” (97). However, these findings represent only a small percentage of children with KS, as around 10% are diagnosed before puberty (98). The remaining 90% may exhibit a less pronounced developmental phenotype.

Current management guidelines recommend assessment via physical examination, bone status, psychological counseling, and speech and language therapy “if needed” (17). Early diagnosis of psychiatric comorbidities and intervention may improve HR-QoL outcomes. Autism research has found that earlier diagnosis and interventions enhanced patient outcomes in children aged 4 to 5 years (99). Research also found poorer education attainment to be associated with adverse mental health (100). People with KS were associated with more significant autism symptoms, poorer education, and increased risk of psychiatric comorbidities, alongside a reduced HR-QoL. Stricter referrals for children diagnosed with KS to psychological and behavioral health services could improve HR-QoL due to early and condition-specific interventions.

Adolescents (aged 10-19 years) and those transitioning to adulthood require individualized care plans that often involve monitoring spermatogenesis, the function of the hypothalamic-pituitary-testicular axis, cognition, and psychological comorbidities. TRT becomes a key consideration during adolescence in cases of delayed or stalled puberty and hypogonadism. Fertility interventions, such as micro-TESE and cryopreservation, warrant further discussion. While fertility interventions are likely to be undertaken in adulthood, earlier discussions when appropriate may provide greater clarity for patients and their families (17, 24, 25).

Health-care providers must ensure a safe and effective transition from pediatric to adult care settings. This process often involves new clinicians or services. Research demonstrates improved outcomes when key elements such as appropriate parental involvement, promotion of self-efficacy, and early engagement with adult care teams are incorporated (101).

Best practices for transition care, as outlined in the Got Transition Six Core Elements of Health Care Transition 3.0, offer a robust framework for guiding youth and young adults from pediatric to adult health care. This framework emphasizes transition policy, readiness assessment, tracking and monitoring, transition planning, transfer of care, and integration into adult care systems (GotTransition.org). However, individuals with rare conditions like KS often face unique challenges, including participation in decision-making, clinical knowledge gaps, trust issues, and financial and access barriers (102).

Research into the transition from pediatric to adult services for people with hypogonadism recognizes how poor transitional care influences HR-QoL and long-term physical health, emphasizing good transitional care as an ongoing process rather than a one-time event and supporting collaboration between pediatric and adult health-care providers and open communication with adolescents and their families (103).

The life expectancy for people with KS is up to 2 years shorter, suggesting a possibility of age-related illnesses, although the extent of risk remains unclear. Molecular aging research shows a steeper telomere length decrease over time in people with KS compared to HCs (*P* < .01). However, other biomarkers show no significant differences (20). Due to the preexisting risk of cognitive impairment, osteoporosis, and fractures, future research and guidelines may require adapting to an age-specific phenotype.

## Implications for Research and Clinical Practice

Our review identifies considerable gaps in managing KS, particularly regarding HR-QoL. Research and clinical guidelines must both evolve to better address the multifaceted needs of individuals with KS, especially concerning early diagnosis, multidisciplinary care, the risk profiles of various cancers, and comprehensive physical and psychological support.

Early diagnosis of KS could benefit from a collaborative approach; the inclusion of noninvasive prenatal screening can detect KS early, followed by confirmatory testing. Pediatricians should monitor developmental progress especially on the brink of puberty, while further information from educators can provide valuable insights into speech, motor, and social challenges. Combining medical and educational assessments could lead to earlier identification and referrals to genetic testing.

Research in related conditions, such as autism, suggests early detection can significantly enhance long-term HR-QoL. While specific studies in KS are lacking, the potential benefits of early diagnosis and intervention remain largely unexplored. Future investigations should focus on whether early intervention of physiological comorbidities, TRT, and psychological support can bring about improvements across the lifespan.

A considerable gap exists in understanding the psychological phenotype of KS and its resulting effect on HR-QoL. Further questions arise about current management strategies, diagnosis, and clinical knowledge of the psychological profile within KS. Clinicians need integrated approaches addressing both the physical and psychological aspects of KS. At present, guidelines lack sufficient psychological guidance. It is crucial to integrate mental health support into routine care to help alleviate disease burden.

Epigenetic mechanisms play a role in the phenotypic variability of individuals with KS. Given the significant clinical heterogeneity observed in KS, it is unlikely that a single genetic mechanism explains the observed variability. Recent findings reveal widespread alterations in both autosomes and the X chromosome, resulting in a unique molecular profile. While X chromosome inactivation in KS shares similarities with that in females, gene expression patterns remain closer to those of males. Hypermethylation and hypomethylation may contribute to the observed phenotype, as differentially expressed genes relate to immune response, Wnt-signaling pathways, and neuron development. Emerging research findings point to both new candidate genes, which may modify phenotype, and noncoding genes, which may be involved in X chromosome inactivation in people with KS (10), while further research on testicular pathogenesis has identified a significant number of upregulated genes that escape X-chromosome inactivation, supporting the notion that those genes drive the testicular dysfunction/phenotype in KS. Single-cell RNA-sequencing data from testes of adult men with KS have revealed further evidence that the Sertoli cell is the most affected cell type (104).

Future research using genomic, epigenomic, transcriptomic, and proteomic data could clarify the molecular basis of this heterogeneity. Polygenic risk scores may also aid in assessing individual risks for KS-related conditions, supporting personalized clinical management (105).

Strategies to improve clinical decision-making, evaluate intervention effectiveness, and support further research are vital for optimizing patient care. There is a clear need for condition-specific patient-reported outcome measures (PROMs), as existing PROMs do not fully address the unique challenges face by people with KS. Developing PROMs that capture the lived experience of people with KS would advance clinical practice and research.

## Conclusion

Current guidelines for managing people with KS are limited to one endorsed guideline. There is a clear need for international and interprofessional collaboration to develop consensus documents. Importantly, existing guidelines pay minimal attention to the numerous HR-QoL challenges, overlooking the effect and diagnosis of psychological comorbidities. Guidelines should prioritize enhancing HR-QoL and optimizing physical and mental well-being to ensure that people with KS receive comprehensive care. To achieve this, it is vital to conduct research involving cocreation and active participation of patients and the public at all stages. This approach ensures that health-care professionals and patients contribute to the research process, using validated tools and ultimately allowing patients to have the final say (106).

## Data Availability

This scoping review synthesized data solely from previously published studies identified through our systematic search. All extracted information, including calculated effect sizes, were derived from the reported results in the included peer-reviewed articles. No new empirical data were generated.

## Abbreviations

BMD: bone mineral density
DXA: dual-energy x-ray absorptiometry
FSH: follicle-stimulating hormone
FT4: free thyroxine
HCs: healthy controls
HR-QoL: health-related quality of life
INSL3: insulin-like peptide
KS: Klinefelter syndrome
PROM: patient-reported outcome measures
SIR: standardized incidence ratio
T: testosterone
T2DM: type 2 diabetes mellitus
TESE: testicular sperm extraction
TRT: testosterone replacement therapy
